# Anion–cation synergy enables the formation of ionic liquid-derived adaptive interphases for ultra-stable zinc–metal batteries

**DOI:** 10.1039/d6sc04639h

**Published:** 2026-07-26

**Authors:** Kaisheng Sun, Yanlei Geng, Runan Li, Gaorui Gu, Ningzhi Cao, Chaopeng Zhang, Fangfei Li, Liang Li, Xiaoteng Jia, Danming Chao, Caiyun Wang

**Affiliations:** a Synergetic Extreme Condition High-Pressure Science Center, State Key Laboratory of High Pressure and Superhard Materials, College of Physics, Jilin University 130012 Changchun P. R. China lifangfei@jlu.edu.cn lliang@jlu.edu.cn; b National and Local Joint Engineering Laboratory for Synthetic Technology of High Performance Polymer, College of Chemistry, Jilin University Changchun 130012 China chaodanming@jlu.edu.cn; c State Key Laboratory of Integrated Optoelectronics, College of Electronic Science and Engineering, Jilin University Changchun 130012 China; d Intelligent Polymer Research Institute, AIIM Facility, Innovation Campus, University of Wollongong North Wollongong NSW 2500 Australia caiyun@uow.edu.au

## Abstract

Ionic liquid additives offer a promising strategy for constructing solid electrolyte interphases (SEIs) in zinc–metal batteries (ZMBs), yet the interfacial anion–cation synergy remains poorly understood. Herein, we introduce 1-ethyl-3-methylimidazolium methanesulfonate, which *in situ* forms a hierarchically structured SEI consisting of an inner ion-conductive/electron-insulating ZnS sacrificial layer and an outer potential-responsive dynamic cation–anion adsorption layer (DAL). The ZnS layer originates from the decomposition of methanesulfonate anions, while the DAL consists of electrostatically anchored cations and anions that undergo real-time field-driven rearrangement for uniform Zn deposition/stripping. Additionally, the DAL reconstructs the interfacial hydrogen-bonding network, suppresses side reactions, and dynamically regulates Zn^2+^ adsorption energy, thereby facilitating rapid ion transport. Consequently, Zn//Zn symmetric cells achieve an ultra-long lifespan of 9000 h at 2 mA cm^−2^/1 mAh cm^−2^, while Zn//Cu cells deliver 99.6% average coulombic efficiency. This work elucidates the interfacial behavior of ionic liquid additives and provides new insights for electrolyte design toward ultra-stable ZMBs.

## Abstract 2

Using 1-ethyl-3-methylimidazolium methanesulfonate as an electrolyte additive regulates the solvation structure and constructs an SEI composed of a sacrificial layer and a dynamic adsorption layer, greatly improving zinc battery stability.

## Graphical abstract

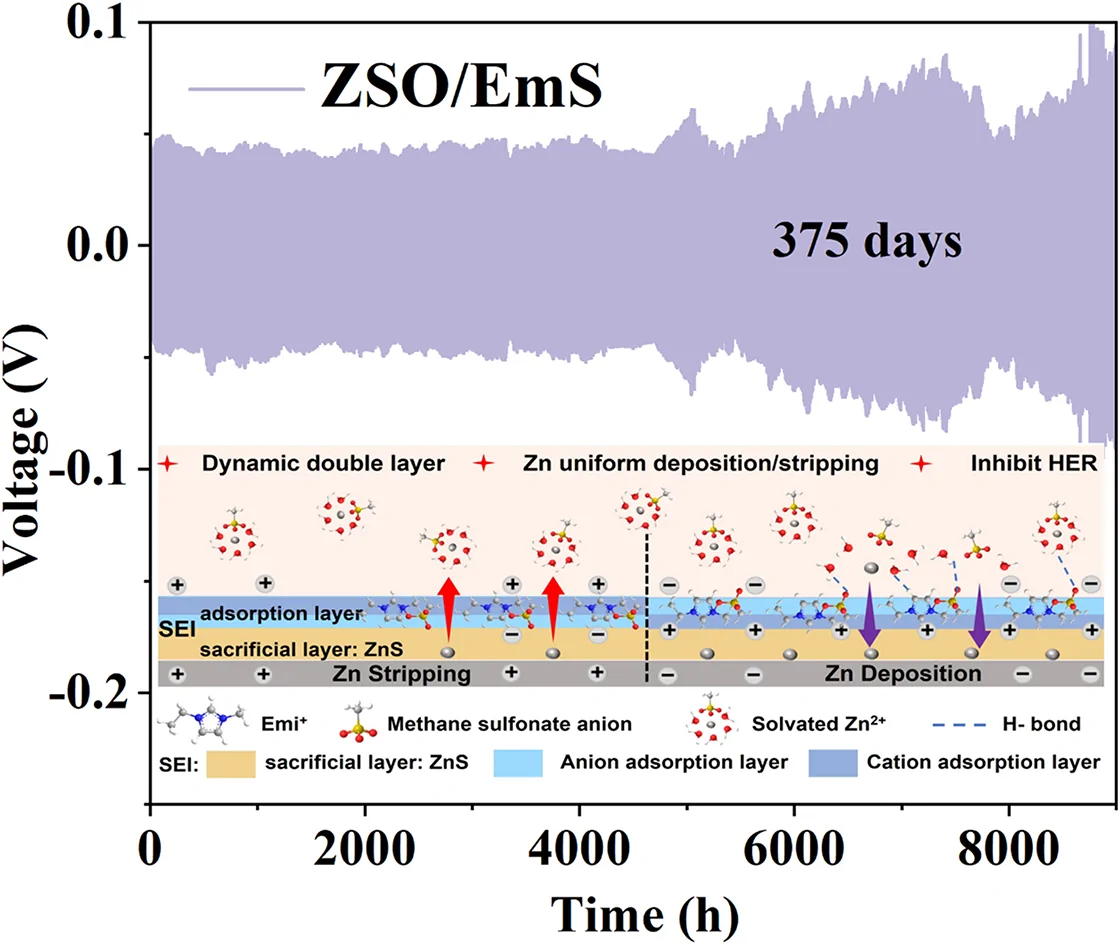

## 1 Introduction

Zinc metal (Zn) is a promising anode for rechargeable batteries due to its high theoretical capacity (820 mAh g^−1^), good safety, and low cost.^1–4^ However, in mildly acidic electrolytes, Zn undergoes parasitic corrosion and forms zinc sulfate hydroxide byproducts, leading to a heterogeneous and unstable solid electrolyte interphase (SEI).^5–8^ This defective interface induces non-uniform Zn deposition, promoting dendrite growth and accelerating interfacial degradation. Meanwhile, interfacial water activity promotes the hydrogen evolution reaction (HER), continuously consuming electrolyte and ultimately causing poor reversibility.^9–11^ To address these challenges, strategies such as electrolyte engineering, surface coating, and alloying have been employed to enhance the stability of Zn and, consequently, the overall performance of Zn–metal batteries (ZMBs).^12–17^ Among these, electrolyte engineering that enables the *in situ* formation of a uniform and stable SEI layer is particularly effective, as it regulates Zn^2+^ flux, homogenizes the electric field, and suppresses interfacial corrosion and side reactions, thereby improving cycling stability.^18,19^

Despite the effectiveness of conventional electrolyte engineering in constructing stable SEIs and improving Zn deposition uniformity,^20–22^ it pays relatively little attention to the Zn stripping process. Ionic liquids (ILs), leveraging cation–anion synergy and interfacial dynamic adsorption, hold promise for simultaneously regulating uniform Zn deposition and stripping.^23–28^ However, the influence of potential variations during Zn deposition/stripping on interfacial ion adsorption behavior remains poorly understood. For example, imidazolium cations can form electrostatic shielding layers that suppress corrosion, but this protection is sensitive to potential variation and deteriorates over prolonged cycles.^29^ Moreover, existing studies typically focus either on decomposition-derived SEIs while overlooking adsorption layers, or treat the adsorption layer as a static, electrostatically anchored structure.^30–32^ Static adsorption models overlook the potential-dependent interfacial dynamics, leaving ion behavior largely underexplored. Such limitations hinder a comprehensive understanding of the underlying interfacial mechanisms, impeding further performance enhancement. Constructing an SEI that combines mechanical robustness with dynamic adaptability using ionic liquid additives is crucial for elucidating the synergistic roles of their cations and anions and represents a key strategy for enhancing the interfacial reversibility of zinc anodes.

Here, we present a strategy utilizing 1-ethyl-3-methylimidazolium methanesulfonate to simultaneously enable *in situ* SEI formation and regulate the solvent structure, guided by theoretical calculations, thereby enhancing the performance of ZMBs. Specifically, methanesulfonate anions decompose at the interface to form an inner ZnS layer with ion-conducting and electron-insulating characteristics. The adsorbed 1-ethyl-3-methylimidazolium cations (Emi^+^) and methanesulfonate anions assemble into an external dynamic cation–anion adsorption layer (DAL) *via* electrostatic interactions. This layer undergoes real-time field-driven rearrangement, thereby achieving uniform Zn deposition and stripping. In addition, interfacial hydrogen bonding and regulated solvation structures suppress water-induced side reactions. Theoretical calculations reveal a reduced Zn^2+^ desolvation energy barrier. Benefiting from this synergistic interfacial regulation, the battery achieves an ultralong cycle life: stable operation for 9000 h at 2 mA cm^−2^/1 mAh cm^−2^ and over 5000 cycles with an average coulombic efficiency of 99.6%. This work elucidates the cation–anion synergy in IL additives, providing mechanistic insights to guide the rational design of electrolytes for high-performance ZMBs.

## 2 Results and discussion

### 2.1. Modulating effects of ZSO/IL electrolytes

**Fig. 1:**
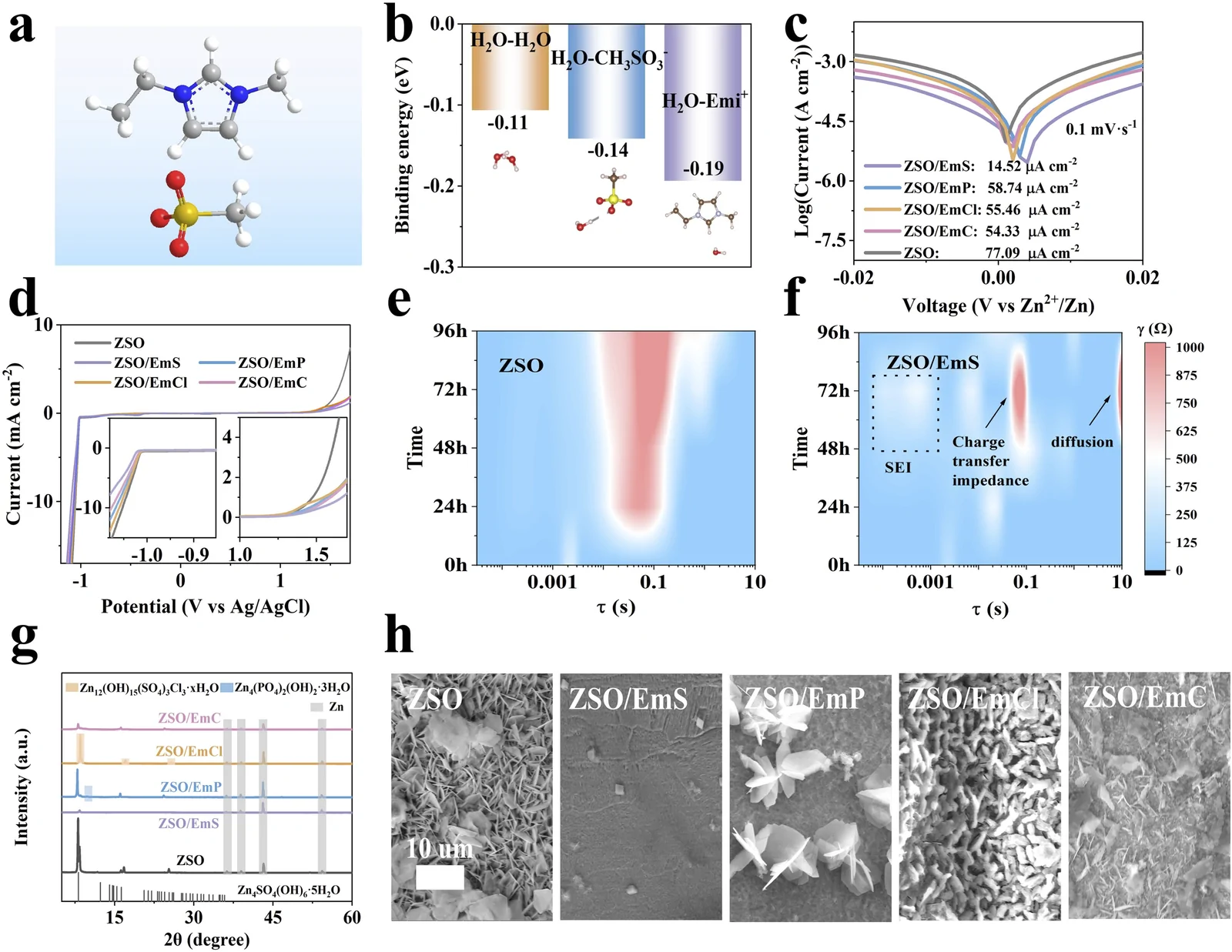
Modulating effects of different electrolytes for stabilising zinc anodes. (a) Molecular configuration of 1-ethyl-3-methylimidazolium methanesulfonate. (b) Binding energies of H_2_O–H_2_O, H_2_O-1-ethyl-3-methylimidazolium, and H_2_O-methanesulfonate interactions. (c) Tafel plots obtained using a three-electrode system for zinc anodes in different electrolytes illustrating the corrosion behaviour. (d) Linear scanning *I*–*V* curves revealing the electrochemical stability window. DRT analysis of Zn//Zn symmetric cells in (e) ZnSO_4_ and (f) ZnSO_4_/EmS electrolytes based on *operando* EIS evolution during resting. (g) XRD patterns and (h) SEM images of Zn metal after 7 days' immersion in different electrolytes.

Four 1-ethyl-3-methylimidazolium-based ionic liquids with distinct anion chemistries (Fig. 1a and S1) were selected as electrolyte additives to suppress Zn corrosion under both operational and static conditions. Ionic liquids (ILs) typically adsorb on the zinc metal surface, forming a protective layer that blocks corrosive species such as water and oxygen.^33,34^ This barrier effect originates from intermolecular interactions, as quantified by binding energy calculations. The IL–H_2_O binding energy (H_2_O-CH_3_SO_3_^−^: −0.14 eV; H_2_O-Emi^+^: −0.19 eV) is stronger than the H_2_O–H_2_O interaction (−0.11 eV) (Fig. 1b), which disrupts the hydrogen-bonding network and effectively excludes water from the interface, thereby mitigating aqueous corrosion.^35,36^ Tafel analysis (Fig. 1c) reveals that the ZSO/EmS (2 M ZnSO_4_ + 0.05 M 1-ethyl-3-methylimidazolium methanesulfonate) electrolyte presents a positive shift in corrosion potential (4.0 mV) and a reduced corrosion current density (14.52 µA cm^−2^), indicating improved corrosion resistance. Linear sweep voltammetry measurements further reveal an expanded electrochemical stability window (ESW) of 2.35 V. This superior performance is attributed to an ionic liquid-derived protective layer that effectively suppresses parasitic reactions like hydrogen evolution. Furthermore, ionic conductivity (Fig. S2) and Zn^2+^ transference number (Fig. S3) measurements reveal that the IL additive optimizes the electrolyte structure and promotes efficient Zn^2+^ transport.

To elucidate the corrosion behavior under static conditions, the impedance evolution of Zn//Zn symmetric cells was analyzed using the distribution of relaxation times (DRT) technique.^37^ The characteristic DRT peaks correspond to the SEI (10^−5^–10^−3^ s), charge transfer resistance (10^−3^–1.0 s), and ion diffusion (1.0–0^2^ s), respectively. As shown in Fig. 1e, the impedance of the conventional ZSO electrolyte increased continuously with standing time, indicative of progressive accumulation of corrosion byproducts. In contrast, the ZSO/EmS electrolyte (Fig. 1f) exhibited a distinct trend: the impedance initially increased, reaching a maximum at ∼72 h, and subsequently decreased. The initial rise can be attributed to rapid but disordered adsorption of ionic liquid molecules on the Zn surface, forming a compact but poorly ion-conductive interfacial barrier that temporarily impedes Zn^2+^ transport. With prolonged standing, these molecules undergo directional reorganization (*e.g.*, negatively charged groups orient to the Zn surface and positively charged groups orient toward the electrolyte).^38^ This adaptive structure not only facilitates Zn^2+^ transport but also continuously suppresses side reactions, thereby significantly reducing charge transfer resistance. A similar impedance evolution (initial increase followed by decrease) was observed for ZSO/EmP (2 M ZnSO_4_ + 0.05 M 1-ethyl-3-methylimidazolium dimethyl phosphate) and ZSO/EmC (2 M ZnSO_4_ + 0.05 M 1-ethyl-3-methylimidazolium acetate), in contrast to a continuous increase for ZSO/EmCl (2 M ZnSO_4_ + 0.05 M 1-ethyl-3-methylimidazolium perchlorate) (Fig. S4 and S5). This discrepancy originates from anion chemistry: perchlorate, with weak coordination, strong hydrophilicity, and low decomposition tendency, hinders the formation of ion-conducting and adaptive interfaces, leading to continuous impedance growth. This highlights the critical role of anion species in governing the dynamic self-optimization of the SEI. The ionic conductivity of ZnS-SEI was estimated to be 6.4 × 10^−2^ mS cm^−1^ (Fig. S6).

To visually evaluate the corrosion inhibition effect of electrolyte additives, Zn foil was immersed in different electrolytes for 7 days, followed by XRD and SEM analysis (Fig. 1g and h). A pronounced peak corresponding to Zn_4_SO_4_(OH)_6_·5H_2_O was observed in the ZSO, indicating severe corrosion. In contrast, this peak was weakened in the ZSO/EmP and ZSO/EmCl systems, accompanied by the emergence of new phases (*i.e.*, Zn_4_(PO_4_)_2_(OH)_2_·3H_2_O and Zn_12_(OH)_15_(SO_4_)_3_Cl_3_·*x*H_2_O), suggesting altered corrosion pathways. Notably, by-product signals were largely suppressed in ZSO/EmC and ZSO/EmS, demonstrating effective corrosion inhibition by the ionic liquid additives. SEM morphology analysis further confirmed these findings. The Zn surface in ZSO was covered with dense flake-like products, whereas their formation in ZSO/EmP and ZSO/EmC was significantly reduced. In contrast, the Zn surface in ZSO/EmCl exhibited rod-like structures, suggesting the formation of different by-products. Remarkably, in ZSO/EmS, the Zn surface remained relatively smooth, confirming its superior corrosion resistance. These morphological observations are consistent with the XRD analysis.

### 2.2. SEI structural characterization

**Fig. 2:**
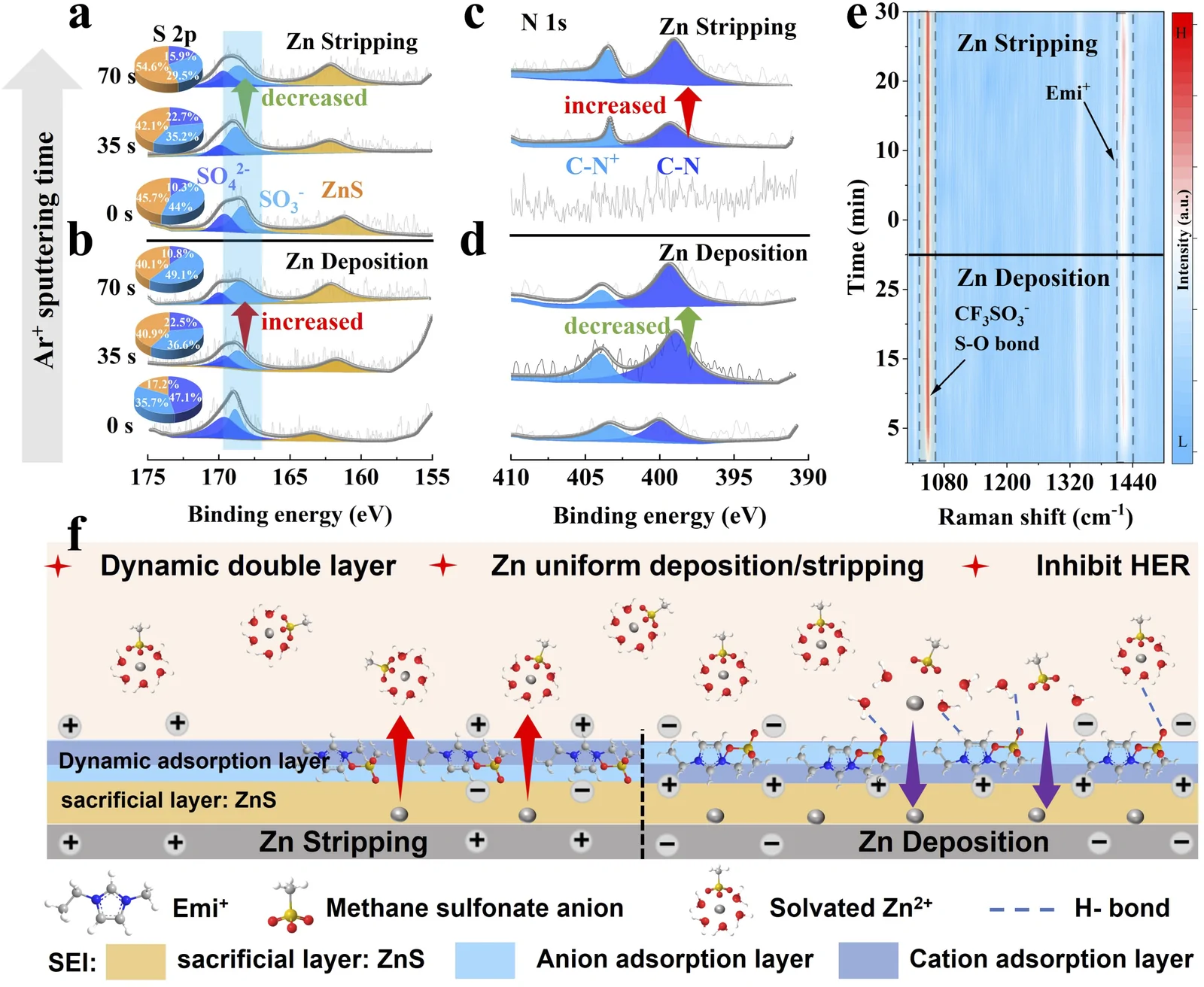
Structural properties of the *in situ* formed SEI. High-resolution XPS spectra during Zn stripping and Zn deposition: (a and b) S 2p and (c and d) N 1s. (e) *In situ* Raman spectra of the electrode in the ZSO/EmS electrolyte. (f) Schematic illustration of the SEI structure.

To investigate the composition and dynamic evolution of the electrode–electrolyte interface, we performed XPS analysis with varying Ar^+^ sputtering times on the SEI formed in ZSO/EmS electrolyte under Zn stripping and deposition conditions. The S 2p spectrum can be deconvoluted into characteristic peaks of SO_4_^2−^ (169.7 eV), SO_3_^−^ (168.7 eV), and ZnS (162.1 eV) (Fig. 2a and b).^29^ The ZnS content intensified with sputtering time (stripping: 45.7–54.6%; deposition: 17.2–40.1%), indicating its location within the inner SEI and confirming its origin from the decomposition of CH_3_SO_3_^−^ species adsorbed on the Zn surface. Due to its high ionic conductivity and electronic insulation, the ZnS layer effectively suppressed the further decomposition of subsequently adsorbed ions. Accordingly, the SEI can be described as a bilayer structure comprising an inner sacrificial ZnS layer and an outer dynamic cation–anion adsorption layer (DAL) formed by adsorbed ions. The SO_3_^2−^ signal associated with CH_3_SO_3_^−^ decreased with sputtering during Zn stripping (44.0–29.52%) but increased during Zn deposition (35.7–49.1%), suggesting a potential-dependent redistribution of adsorbed CH_3_SO_3_^−^ species at the Zn/electrolyte interface. To further verify this behavior, N 1s and C 1s spectra were analyzed. Nitrogen, derived from the imidazolium-based cation, can be fitted to C–N^+^ (403.3 eV) and C–N (399.4 eV) species (Fig. 2c and d).^39^ Its intensity increased with sputtering during Zn stripping and decreased during deposition. A similar trend was observed for C 1s (Fig. S7).

*In situ* Raman spectroscopy was further employed to monitor the evolution of the interfacial adsorption layer during battery operation. During the initial activation stage (0–5 min), the characteristic peaks of CH_3_SO_3_^−^ (S–O bond: ≈1048 cm^−1^) and Emi^+^ (≈1420 cm^−1^) increased significantly, confirming the formation of the adsorption layer. Moreover, the Emi^+^ peak intensity differed markedly between Zn deposition and stripping states, with a higher intensity observed during stripping (Fig. 2e). These results collectively confirm the dynamic and reversible restructuring of the outer DAL in response to the applied potential.

In summary, the *in situ* formed SEI consists of an inner sacrificial layer and an outer dynamic cation–anion adsorption layer. The outer layer can be further divided into cationic and anionic sublayers with potential-dependent spatial distributions: cations preferentially enrich the outermost layer during Zn stripping, whereas anions become dominant during Zn deposition. This dynamic arrangement enables the SEI to adapt to evolving interfacial conditions and retain stability. A schematic illustration of the proposed SEI structure and its dynamic evolution is shown in Fig. 2g.

### 2.3. Electrochemical performances

**Fig. 3:**
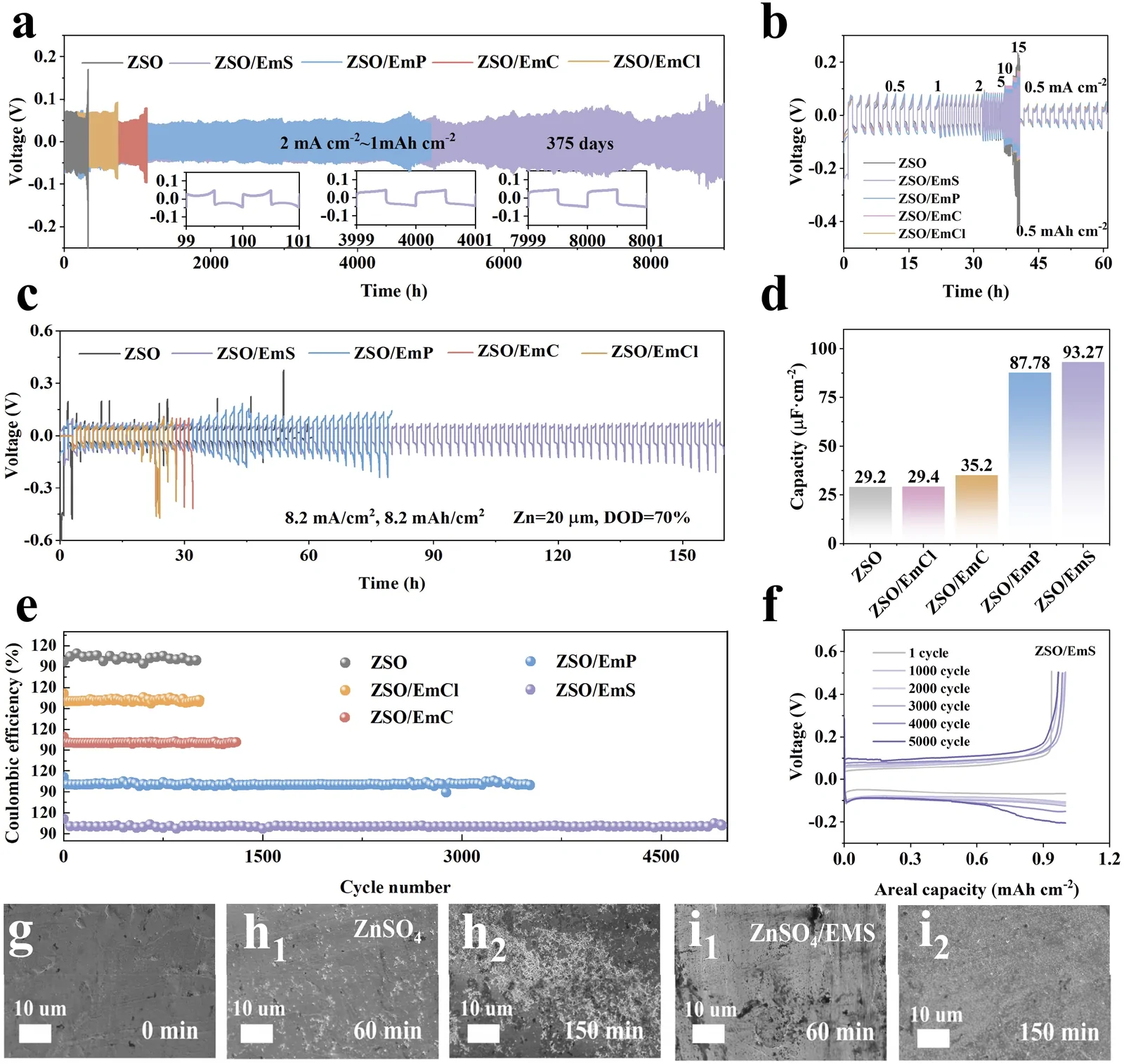
Electrochemical performance of symmetric and full cells in different electrolytes. (a) The cycle performance of Zn//Zn symmetric cells at a current density of 2 mA cm^−2^ and capacity of 1 mAh cm^−2^. (b) Rate performance of Zn//Zn symmetric cells. (c) Cycling stability of Zn//Zn symmetric cells at 70% depth of discharge. (d) Double-layer capacitance at Zn electrode interfaces in different electrolytes. (e) Coulombic efficiency of Zn//Cu asymmetric cells. (f) Voltage curves of Zn/Cu batteries at different numbers of cycles. (g) SEM images of Zn foil and deposited Zn layers in (h) ZSO and (i) ZSO/EmS electrolytes at a current density of 1.0 mA cm^−2^ for 60 min and 150 min.

The effects of different IL additives on the Zn anode performance in ZSO-based electrolytes were systematically evaluated. The effect of additive concentration was first examined (Fig. S8), with 0.05 M EmS showing the best electrochemical stability. At 0.02 M, the ionic liquid supply at the interface is insufficient to establish an effective adsorption layer, whereas 0.2 M increased electrolyte viscosity and impeded Zn^2+^ transport, leading to impaired reaction kinetics. As shown in Fig. 3a, the introduction of EmS enabled the Zn//Zn symmetric cell to achieve a cycle life of 9000 hours at 2 mA cm^−2^/1 mAh cm^−2^, significantly outperforming the pristine ZSO electrolyte, which short-circuited after only 320 hours (∼28-fold improvement). Notably, the EmS-based system surpasses previously reported electrolyte additives in cycling performance (Table S1). Other additive systems (ZSO/EmP, ZSO/EmC, and ZSO/EmCl) also exhibited enhanced stability, with cycle lives of 5000, 1114 h, and 726 h, respectively. Rate capability tests further confirmed this advantage (Fig. 3b). In the ZSO/EmS system, the cell showed the smallest overpotential and the best stability across all current densities, in sharp contrast to the significantly increased overpotentials in the ZSO system, especially at high current densities. Moreover, even under extreme conditions with 70% depth of discharge (DOD), the ZSO/EmS cell remained in stable operation for 160 hours (Fig. 3c), demonstrating robust interfacial stability. These notable improvements originate from the adaptive SEI that effectively enhances the reversibility of zinc plating/stripping. The double-layer capacitance at the Zn surface can serve as an indicator of Zn^2+^ deposition sites. The ZSO/EmS cell exhibited the highest double-layer capacitance (Fig. 3d and S9–S13), indicative of increased accessible deposition sites. This is attributed to the synergistic effect of the inner ZnS layer and the outer EmS adsorption layer, which together homogenize the surface electric field, thereby promoting uniform Zn^2+^ adsorption. Furthermore, EmS molecules within the electric double layer interact with interfacial water molecules through hydrogen bonding, preventing direct contact with the Zn surface and increasing available active sites. Together, these effects enabled the *in situ*-formed functionally adaptive SEI to promote uniform and dense Zn deposition.

The effect of IL additives on Zn deposition/stripping behavior was further investigated in Zn//Cu asymmetric cells through coulombic efficiency (CE) tests at 5 mA cm^−2^ and 1 mAh cm^−2^ (Fig. 3e and f). The cell using ZSO electrolyte exhibited pronounced performance decay after 500 cycles, accompanied by significant CE fluctuations, indicating poor reversibility due to side reactions and zinc dendrite growth. In contrast, the addition of IL additives generally improved cycling stability. Particularly in the ZSO/EmS system, the cell achieved up to 5000 stable cycles with an average CE of 99.6%, demonstrating that EmS effectively suppresses side reactions and enables highly reversible Zn plating/stripping. To elucidate the origin of cycling stability from a microstructural perspective, the surface of the Zn electrode was examined after deposition for 0, 60, and 150 min at a current density of 1.0 mA cm^−2^ in ZSO and ZSO/EmS electrolytes. In the ZSO electrolyte, irregular protrusions progressively developed with deposition time, driven by dendrite growth and side reaction-induced corrosion. In contrast, the Zn surface in the ZSO/EmS electrolyte retained a smooth and uniform morphology, directly demonstrating the effectiveness of EmS in promoting uniform zinc deposition. Cross-sectional EDS analysis revealed a uniform ZnS layer with an average thickness of ∼25 nm on the Zn foil (Fig. S14).

### 2.4. Theoretical calculations

**Fig. 4:**
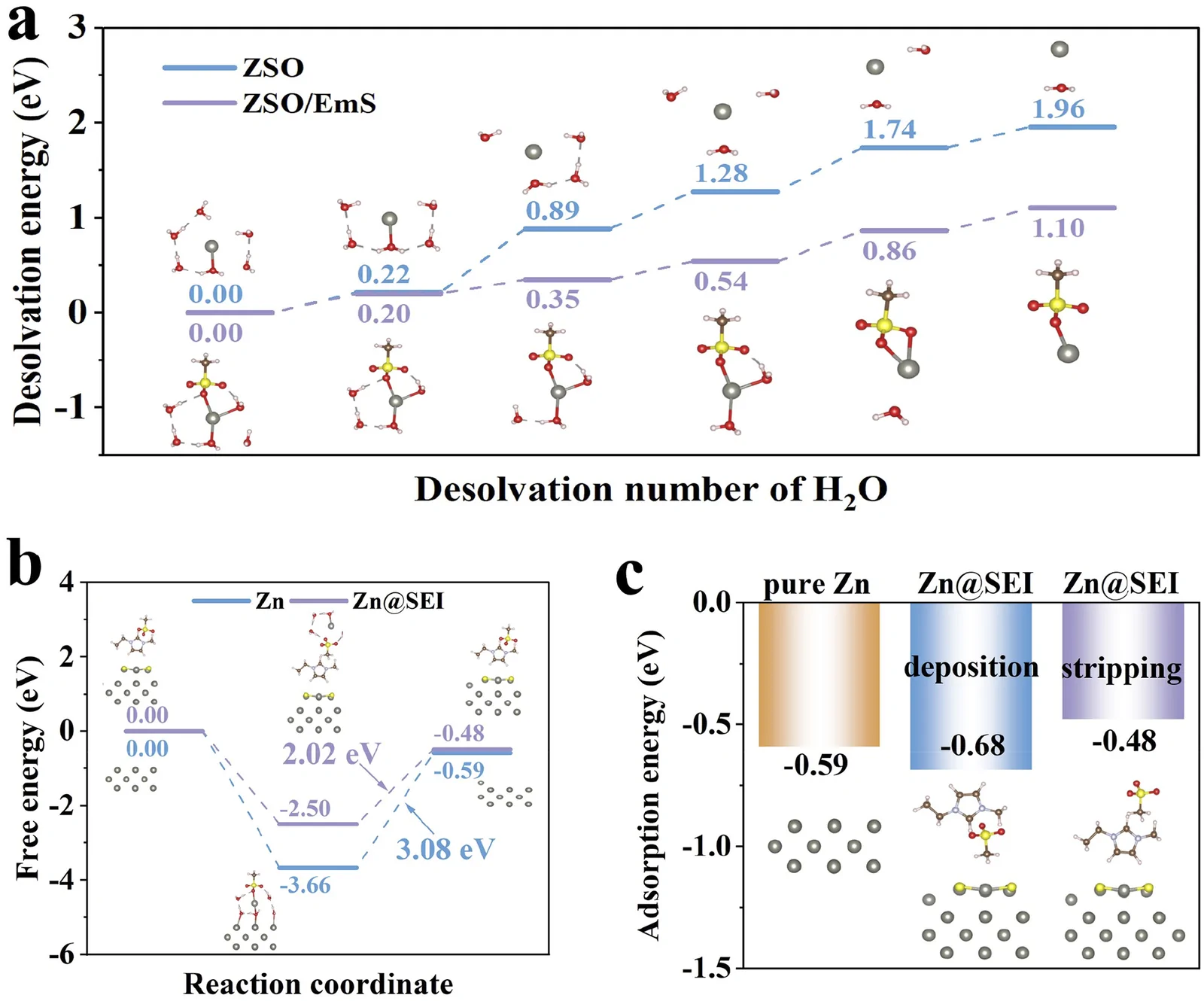
DFT analysis of electrolytes and interfaces. (a) The energy required to sequentially remove coordinated water from Zn^2+^ in ZSO and ZSO/EmS electrolytes. (b) Desolvation energy barrier. (c) Adsorption energies of Zn^2+^ on pristine Zn and SEI-covered Zn (Zn@SEI).

Fig. 4a quantifies the energy consumed for dissociating each solvent molecule from solvated Zn^2+^ in ZSO and ZSO/EmS electrolytes. The ZSO/EmS system exhibited consistently lower energy consumption at each dissociation step, indicating a weakened coordination between Zn^2+^ and water molecules. This effect is attributed to the effective modulation of the Zn^2+^ solvation structure by CH_3_SO_3_^−^. To further elucidate interfacial kinetics, computational models were constructed for pristine Zn and dynamic SEI-modified Zn (Zn@SEI). Based on the free energy difference between the adsorbed and deposited states, the desolvation energy barriers of solvated Zn^2+^ were determined (Fig. 4b). The desolvation energy barrier at the Zn@SEI interface was 2.02 eV, significantly lower than 3.08 eV for pristine Zn. This indicates that the SEI layer effectively reduces the desolvation resistance and accelerates the deposition kinetics of Zn^2+^.

Considering the dynamic variation of the adsorption layer structure within the SEI with applied potential, two models corresponding to the Zn deposition and stripping states were constructed, and their effects on the Zn^2+^ adsorption energy were evaluated (Fig. 4c). The adsorption energy of Zn^2+^ on pristine Zn was −0.59 eV. In contrast, at the Zn@SEI interface, the adsorption energy strengthened to −0.68 eV during deposition and weakened to −0.48 eV during stripping. This disparity arises from the potential-dependent adaptive reconstruction of the adsorbed layer: enhanced adsorption during deposition facilitates rapid Zn^2+^ deposition, while weakened adsorption during stripping promotes efficient Zn^2+^ removal. Such dynamic interfacial regulation characteristics not only improve the kinetics of Zn^2+^ deposition/stripping but also enhance interfacial reversibility, ultimately contributing to prolonged cycling life.

### 2.5. Full cell performance

**Fig. 5:**
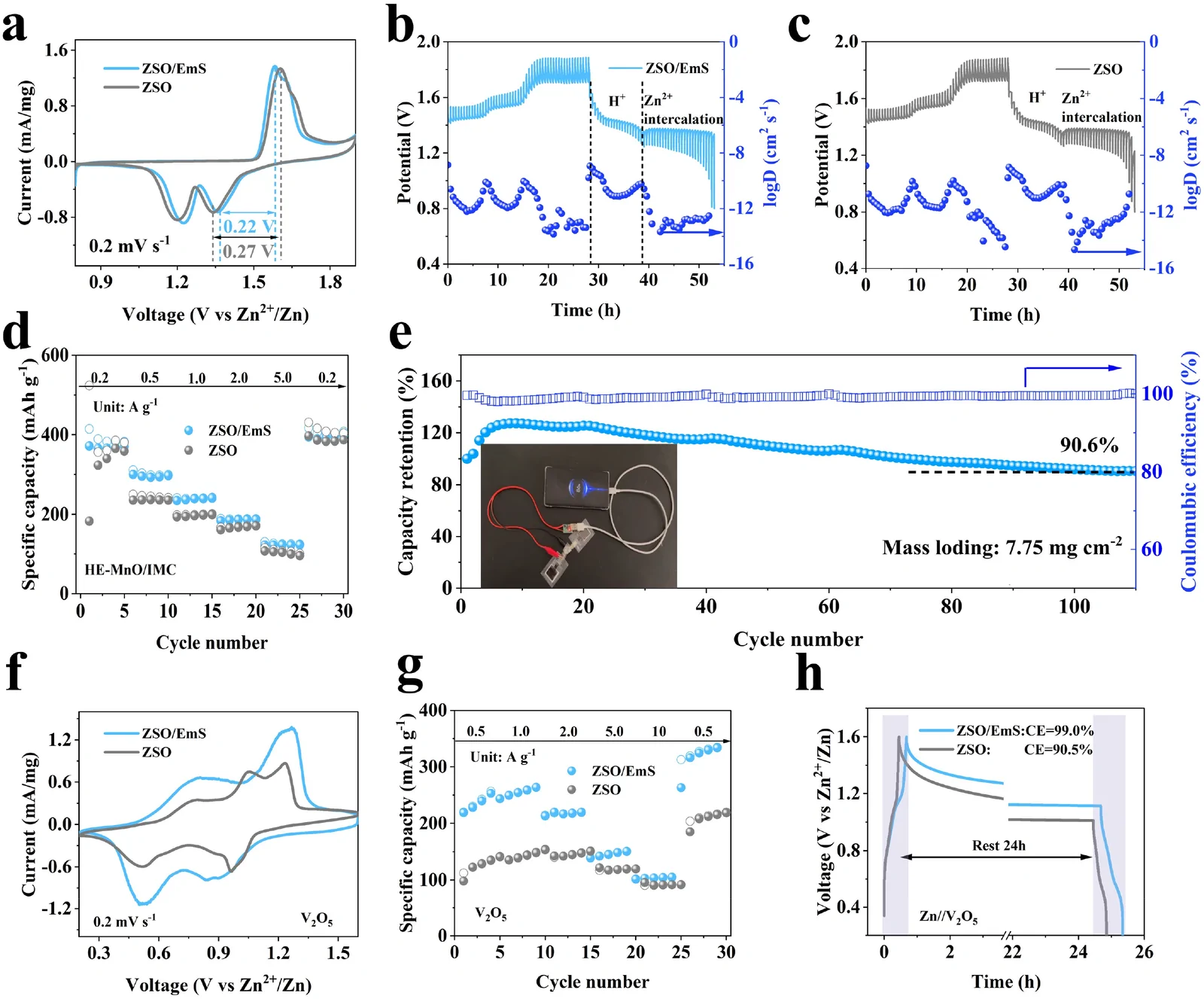
Electrochemical performances of Zn‖HE-MnO/IMC and Zn‖V_2_O_5_ full cells. (a) CV curves of HE-MnO/IMC in ZSO/EmS and ZSO electrolytes at 0.2 mV s^−1^. GITT curves of (b) ZSO/EmS and (c) ZSO. (d) Rate performance of Zn‖HE-MnO/IMC coin cells. (e) Zn‖HE-MnO/IMC pouch cells using a ZSO/EmS electrolyte and photos demonstrating mobile phone charging. (f) CV curves of V_2_O_5_ in ZSO/EmS and ZSO electrolytes at 0.2 mV s^−1^. (g) Rate performance of Zn//V_2_O_5_ coin cells. (h) Self-discharge curve of Zn//V_2_O_5_ cells in ZSO/EmS and ZSO.

To further verify the practical applicability of EmS as an electrolyte additive, its performance was evaluated in a Zn‖HE-MnO/IMC full cell system. The cathode material HE-MnO/IMC was fabricated following the procedures reported and enabled stable operation for 80 cycles at a high mass loading of 5.8 mg cm^−2^.^40^ However, cells using ZSO/EmS and ZSO electrolytes exhibited similar CV curves (Fig. 5a), indicating that the introduction of EmS does not alter the fundamental energy storage mechanism. The reduced potential difference between redox peaks in ZSO/EmS (0.22 V) compared to ZSO (0.27 V) suggests reduced polarization and enhanced reaction kinetics. Based on GITT measurements of charge carrier diffusion (Fig. 5b and c), the ZSO/EmS system (ranging from −8.87 to −13.68) is superior to the ZSO system (ranging from −8.85 to −14.67). This result further confirms that EmS effectively enhances ion transport, particularly facilitating the diffusion of Zn^2+^. Moreover, the cell with ZSO/EmS electrolyte exhibited superior rate performance and higher discharge specific capacity across various current densities (Fig. 5d). It was assembled into a pouch cell to assess its practical potential. As shown in Fig. 5e, the Zn‖HE-MnO/IMC pouch cell exhibited an activation phenomenon with gradually increasing capacity during the first 10 cycles at 2.0 mA cm^−2^, with an areal capacity of 4.9 mAh and an N/P ratio of 4.8. After 110 cycles, the capacity retention remains above 90% with a high coulombic efficiency. Moreover, the pouch cell can charge a mobile phone battery, further confirming its good practical prospects.

To further verify the universality of the EmS additive, commercial V_2_O_5_ was selected as the cathode material. As shown in the CV curves (Fig. 5f), the integrated area in the ZSO/EmS electrolyte is significantly larger than that in the bare ZSO, confirming the enhanced energy storage performance. Moreover, the Zn‖V_2_O_5_ cell with EmS exhibits superior rate capability compared to the control (Fig. 5g). The self-discharge behavior was further evaluated by charging the cell to 1.60 V, resting for 24 h, and discharging to 0.2 V. The capacity retention in the ZSO/EmS system reached 99.0%, higher than that in the ZSO system (90.5%, Fig. 5h), indicating that EmS effectively suppresses side reactions during storage.

## 3 Conclusions

In summary, this work introduces an ionic liquid additive strategy to effectively address the sluggish Zn^2+^ transport kinetics and interfacial instability in zinc–metal batteries. By incorporating 1-ethyl-3-methylimidazolium methanesulfonate, a functionally graded SEI was constructed *in situ* on the Zn anode, consisting of an inner ZnS layer and an outer dynamic cation–anion adsorption layer. This structure enables uniform Zn^2+^ deposition and stripping and suppresses side reactions. DFT simulations further reveal that methanesulfonate anions regulate the Zn^2+^ solvation structure and lower the desolvation energy barrier, while the adaptive SEI enhances Zn^2+^ adsorption and transport, collectively accelerating interfacial kinetics. As a result, Zn//Zn symmetric cells achieve an ultralong cycle life of 9000 h, an average coulombic efficiency of 99.6%, and stable operation at a 70% depth of discharge. Moreover, Zn–Mn pouch cells with a high mass loading of 7.75 mg cm^−2^ exhibit excellent cycling stability, highlighting strong potential for practical applications.

## Author contributions

K. S.: writing – review and editing, writing – original draft, validation, software, methodology, investigation, formal analysis, conceptualization. Y. G.: validation, supervision, software. R. L., G. G., N. C., C. Z., L. L., F. L., and X. J.: data acquisition, software, and formal analysis. D. C.: methodology, funding acquisition, supervision, and writing – review and editing. C. W.: methodology and writing – review and editing.

## Conflicts of interest

There are no conflicts of interest to declare.

## Supplementary Material

SC-017-D6SC04639H-s001

## Data Availability

The data used for this study are available from the corresponding authors upon request. Supplementary information (SI): experimental and computational details and SI figures and tables. See DOI: https://doi.org/10.1039/d6sc04639h.
