# Supplementary material for: Anion–cation synergy enables the formation of ionic liquid-derived adaptive interphases for ultra-stable zinc–metal batteries
Source: Chem Sci. 2026 Jul 26;17(36):17409–17. doi: 10.1039/d6sc04639h (PMC13425459; doi:10.1039/d6sc04639h)
Supplement: Supplementary file 1 [file SC-017-D6SC04639H-s001.pdf]

Supplementary Information (SI) for Chemical Science.
This journal is © The Royal Society of Chemistry 2026

Supporting Information

Anion–Cation Synergy Enables Ionic Liquid–Derived Adaptive Interphases for Ultra-Stable Zinc Metal Batteries

*Kaisheng Sun, †[a] [b] Yanlei Geng, †[a], Runan Li, [c] Gaorui Gu, [b] Ningzhi Cao, [b] Chaopeng Zhang, [b] Fangfei Li, *[a] Liang Li, *[a] Xiaoteng Jia, [c] Danming Chao, *[b] Caiyun Wang, *[d]*

[a]Synergetic Extreme Condition High-Pressure Science Center, State Key Laboratory of High Pressure and Superhard Materials, College of Physics, Jilin University, Changchun, 130012, China

[b]National and Local Joint Engineering Laboratory for Synthetic Technology of High Performance Polymer, College of Chemistry, Jilin University, Changchun 130012, China

[c]State Key Laboratory of Integrated Optoelectronics, College of Electronic Science and Engineering, Jilin University, Changchun, 130012, China

[d]Intelligent Polymer Research Institute, AIIM Facility, Innovation Campus, University of Wollongong, North Wollongong, NSW 2500, Australia

*Corresponding Author

†These authors contributed equally

Experimental section

Type of Electrolyte:

The electrolyte for the Zn//Zn symmetric cell consists of 2 M $ZnSO_4$ with 0.05 M of either 1-ethyl-3-methylimidazolium methanesulfonate (EmS), 1-ethyl-3-methylimidazolium dimethyl phosphate (EmP), 1-ethyl-3-methylimidazolium acetate (EmC), or 1-ethyl-3-methylimidazolium perchlorate (EmCl) added.

The full-cell electrolyte consists of 2 M $ZnSO_4$, 0.2 M $MnSO_4$, and 0.05 M 1-ethyl-3-methylimidazolium methanesulfonate.

Precursor MOF synthesis:

$M(CH_3COO)_2{\cdot}nH_2O$ salts ($Mn(CH_3COO)_2{\cdot}4H_2O$ (2.499 mmol), $Cu(CH_3COO)_2$ (0.24 mmol), $Co(CH_3COO)_2{\cdot}4H_2O$ (0.104 mmol), $Ni(CH_3COO)_2{\cdot}4H_2O$ (0.104 mmol), $Zn(CH_3COO)_2$ (0.208 mmol), $Mg(CH_3COO)_2$ (0.208 mmol)) and polyvinyl pyrrolidone (PVP, 150 mg) were dissolved in a mixed solvent of ethanol/H_2O (50 mL/50 mL) under stirring to form Solution A. Separately, 1,3,5-benzenetricarboxylic acid (H_3BTC, 5.353 mmol) was dissolved in another ethanol/H_2O mixture (50 mL/50 mL) under stirring to form Solution B. Solution B was then slowly added to Solution A under stirring for 1 h. The mixture was allowed to stand at room temperature for 24 h. The resulting precipitate was collected by washing, dried under vacuum at 60 °C for 12 h, yielding (CuCoNiZnMg)Mn-BTC.

Preparation of HE-MnO/IMC Cathode:

HE-MnO/IMC was obtained by calcining precursor MOF, under N_2 at 700 °C for 2 h (heating rate: 5 °C/min).

Electrode preparation and battery assembly:

Coin battery：

The cathode slurry contained active material, Ketjen Black, and PVDF (mass ratio 7:2:1). DMF was added dropwise to the powder mixture under stirring to form a uniform slurry. The slurry was coated onto either a stainless-steel wire mesh (mass loading ~ 1.0 mg cm^{-2}). Coated electrodes were dried at 80 °C for 12 h under vacuum.

CR2032-type coin cells were assembled using 190 μL of electrolyte and whatman glass fiber filters (Grade GF/A) separators. Prior to cell assembly, Zn foils were sequentially polished with 3000-, 5000-, and 7000-grit SiC sandpapers to remove the surface oxide layer and obtain a smooth and flat surface.

Pouch battery：

The slurry was applied to a 2 × 2 cm carbon cloth (with a mass loading of approximately 7~8 mg cm^{-2}). The coated electrode was dried under vacuum at 80 °C for 12 hours.

The battery consists of a zinc foil anode, a cathode, a glass-fiber separator, and an electrolyte.

Electrochemical measurements:

Cyclic Voltammetry (CV): Conducted on a CHI660E electrochemical workstation (China) within a voltage window of 0.8-1.9 V at scan rates ranging from 0.2 mV s^{-1}. Electrochemical Impedance Spectroscopy (EIS): Measured at open circuit voltage using a frequency range of 0.01 Hz to 10,000 Hz and an AC amplitude of 5 mV. The distribution of relaxation times (DRT) was calculated from the EIS data using the open-source MATLAB-based software DRT Tools. Galvanostatic Charge-Discharge (GCD): Performed on a Neware battery testing system (China) between 0.8 V and 1.9 V. The electrochemical stability window (ESW) of the electrolyte was measured by linear

voltammetry using Pt sheets as the working and counter electrodes, and an Ag/AgCl reference electrode, at a scan rate of 2 mV/s. The potential was scanned cathodically from the open-circuit potential (OCP) to -1.1 V and anodically to 1.6 V.

The Zn^{2+} transfer number ($t_{Zn^{2+}}$) was calculated by the following equation:

$$t_{Zn^{2+}} = \frac{I_S(\Delta V - I_0 R_0)}{I_0(\Delta V - I_S R_S)}$$

Where I_0 and R_0 represented the current and resistance before polarization. I_s and R_s represented the current and resistance after polarization. ΔV was corresponded to the applied polarization potential.

Materials characterizations:

X-ray diffraction (XRD): Measured on an X-ray powder diffractometer (Cu Kα radiation, λ = 0.15406 nm) to analyze the crystal structure. X-ray photoelectron spectroscopy (XPS): Conducted on a Thermo ESCALAB 250 XI system using monochromatic Al Kα radiation to determine the elemental compositions and valence states. Scanning electron microscopy (SEM): Performed on a LEO1430VP instrument (Germany) to examine the morphology of materials' nanostructures.

Supplementary Methods

The diffusion coefficients of ions were measured by the GITT method. The diffusion coefficient was as follows:

$$D = \frac{4}{\pi\tau}\left(\frac{m_B V_M}{M_B S}\right)^2\left(\frac{\Delta E_s}{\Delta E_\tau}\right)^2$$

Where τ was the constant current pulse time, the $m_B$and M_B was the mass and molecular mass of MnO. The S was the electrode-electrolyte interface area. The ΔE_s

was the steady-state voltage change caused by the current pulse. The ΔE_τ were voltage changes during the constant current pulse.

Two pieces of stainless steel were used as symmetrical electrodes for ionic conductivity measurements, one piece of glass fiber as a separator, and 70 μL of electrolyte in the CR2032 cell. The ionic conductivity of the electrolytes was calculated as:

$$\delta = \frac{L}{S\,R}$$

Where L is the distance between the 2 pieces of stainless steel, S is the area of the piece of stainless steel, and R is the resistance value (EIS) obtained from EIS.

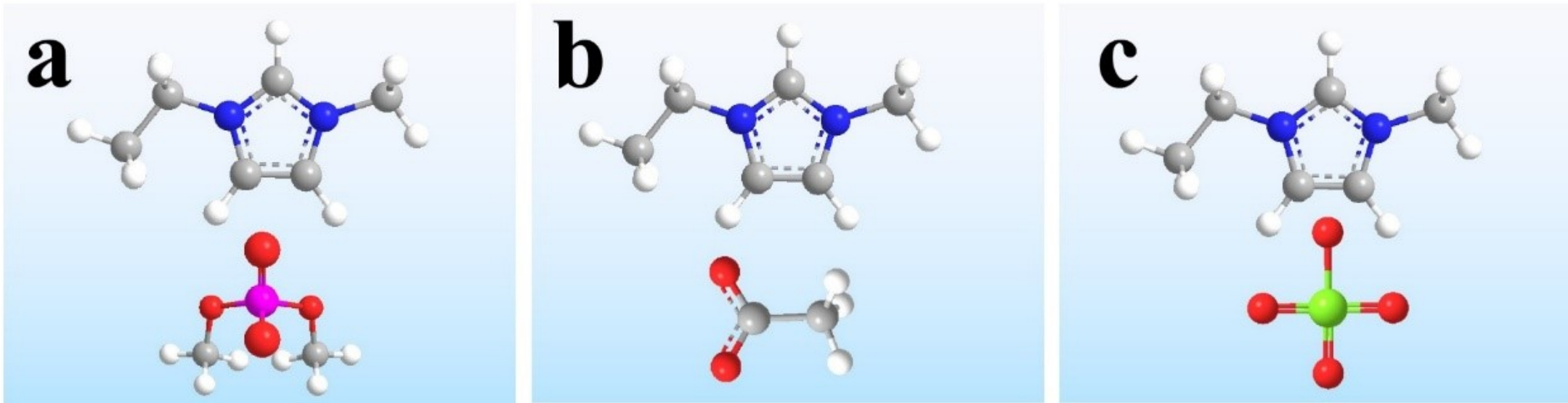

Fig. S1. Molecular configuration of a) 1-ethyl-3-methylimidazolium dimethyl phosphate, b) 1-ethyl-3-methylimidazolium acetate, and c) 1-ethyl-3-methylimidazolium perchlorate.

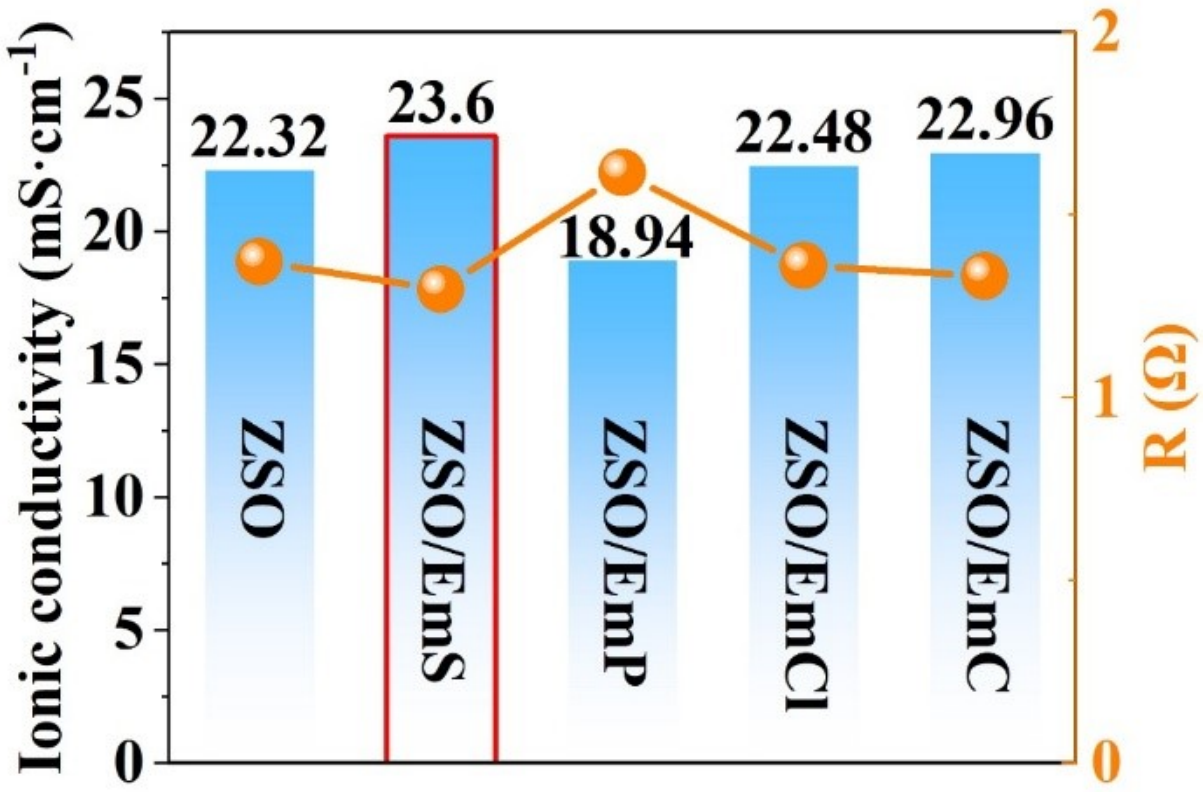

Fig. S2. Ionic conductivity of the electrolyte.

Ionic conductivity measurements show that ZSO/EmS achieves an ionic conductivity of 23.6 mS·cm^{-1}, higher than those of ZSO/EmP (18.94 mS·cm^{-1}), ZSO/EmCl (22.48 mS·cm^{-1}), ZSO/EmC (22.96 mS·cm^{-1}), and pure ZSO electrolyte (22.32 mS·cm^{-1}), indicating that EmS optimizes the electrolyte structure and facilitates rapid ion transport.

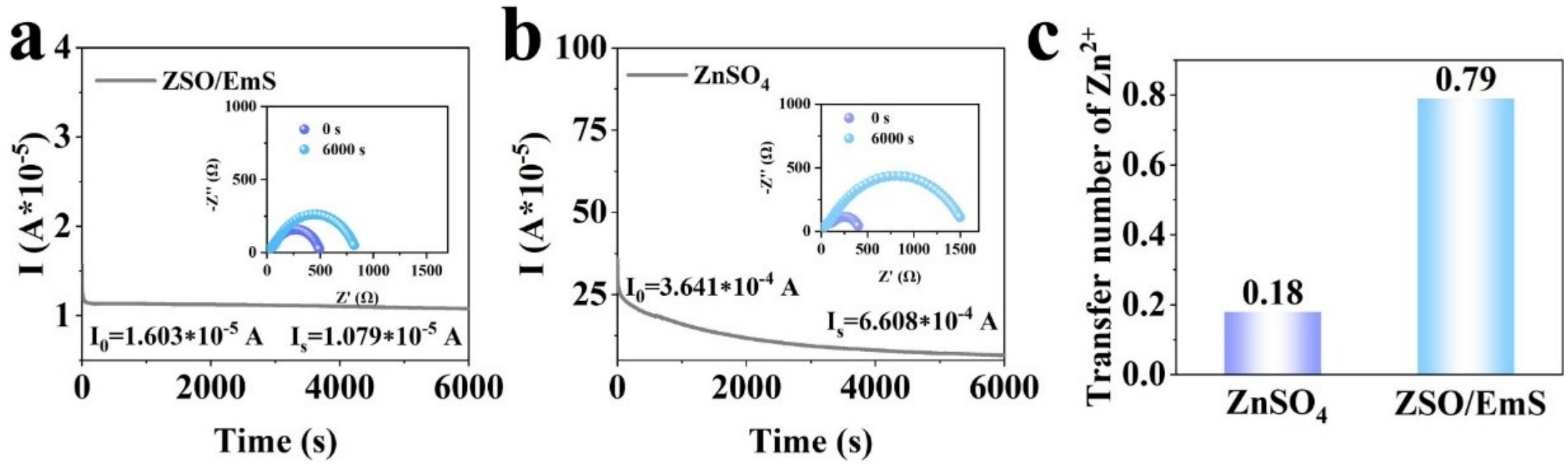

Fig. S3. Calculation of Zn^{2+} migration numbers.

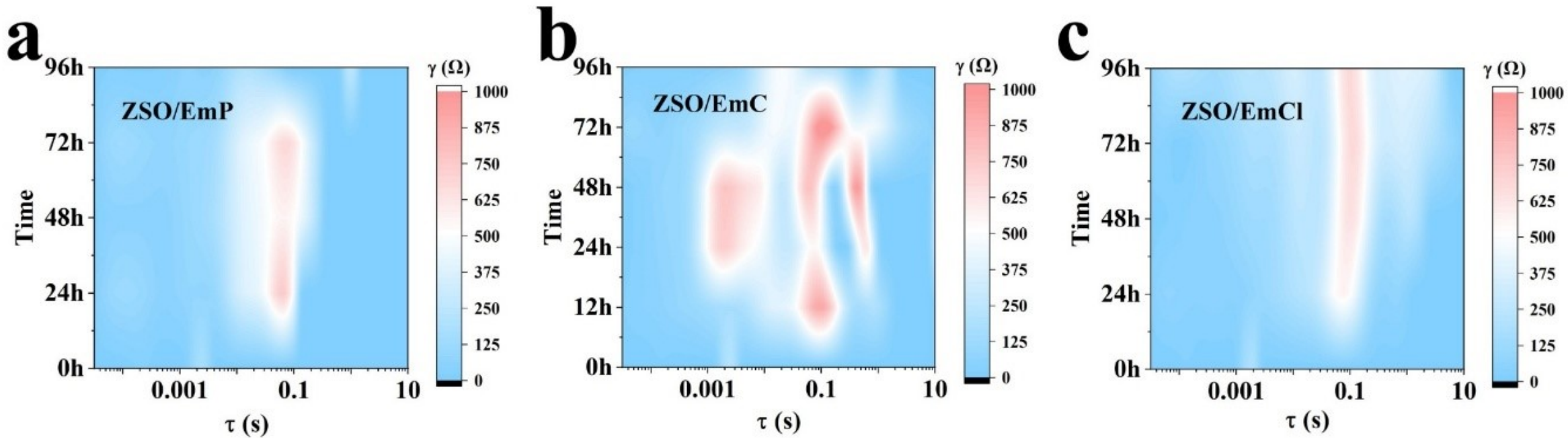

Fig. S4. DRT of analysis of Zn//Zn symmetric cells in a) ZSO/EmP, b) ZSO/EmC, and c) ZSO/EmCl electrolytes by operando EIS evolution during standing.

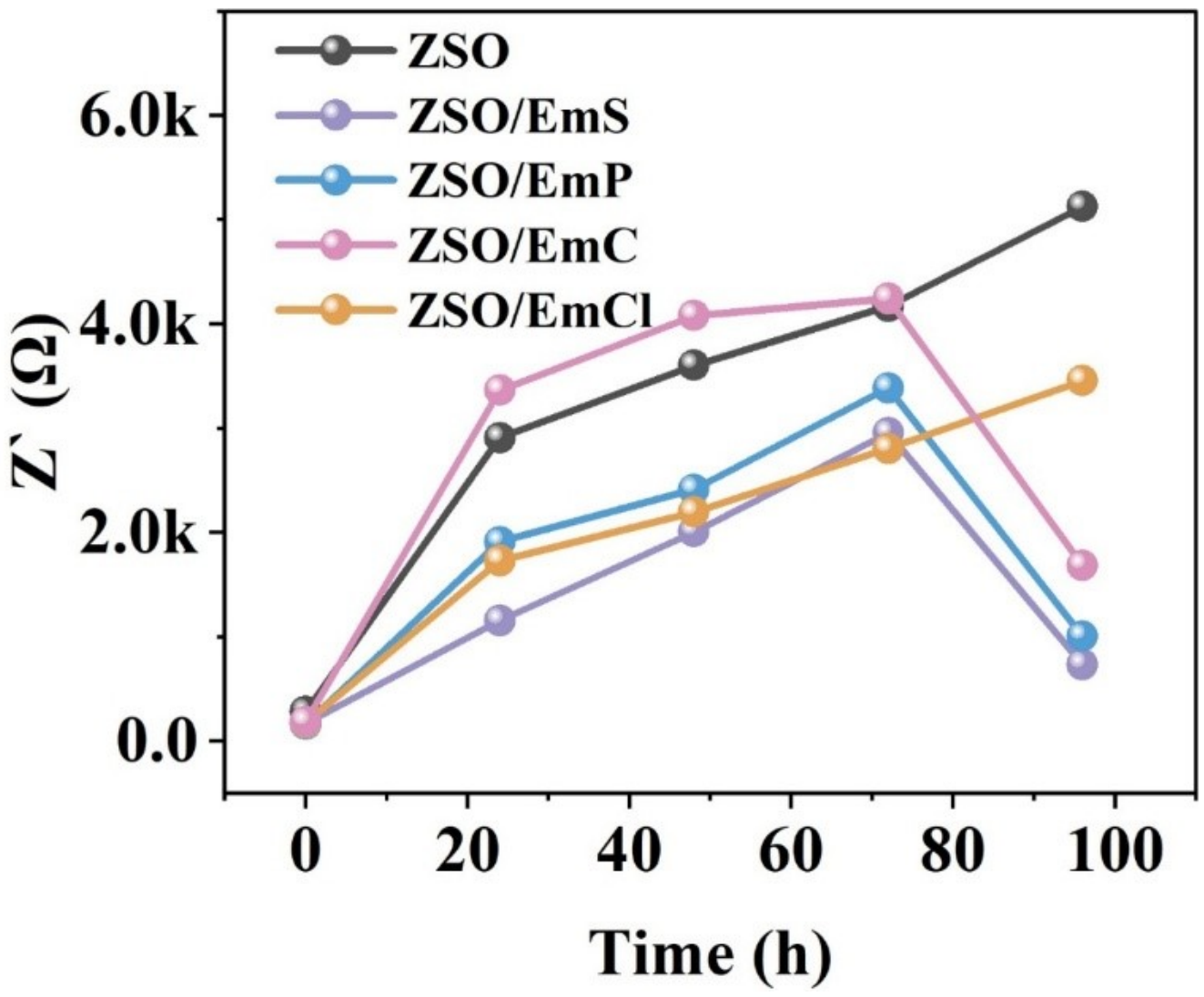

Fig. S5. Variation of impedance with time curve for Zn//Zn batteries in different electrolytes.

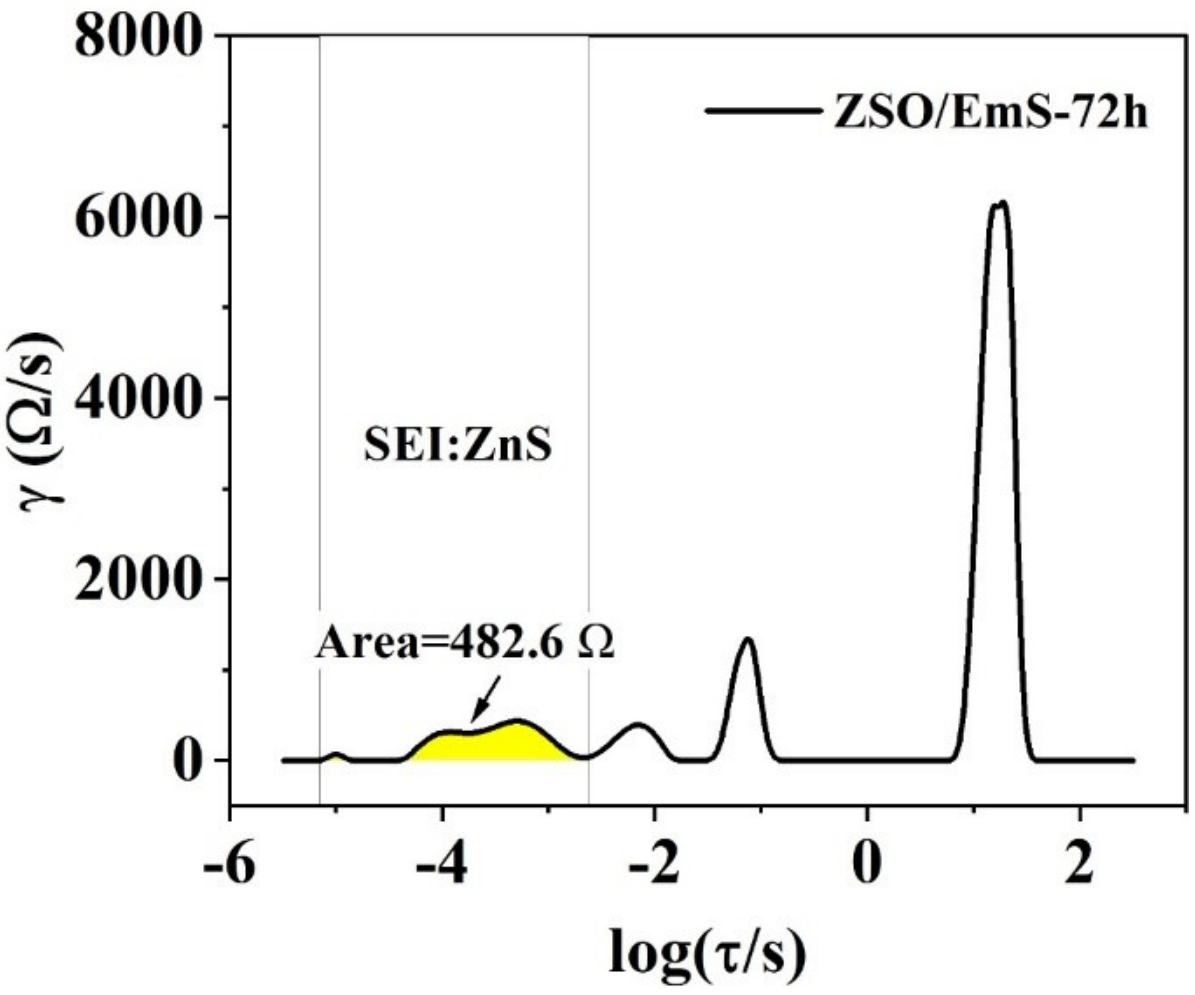

Fig. S6. Impedance and ionic conductivity of ZnS-SEI.

The ZnS-SEI process was distinguished in the time domain by DRT analysis, and the corresponding resistance was determined to be 482.6 Ω by integrating the characteristic peak. The ionic conductivity was then calculated as approximately 6.4×10^{-2} $mS \cdot cm^{-1}$ using the formula $\sigma = L/(S \cdot R)$.

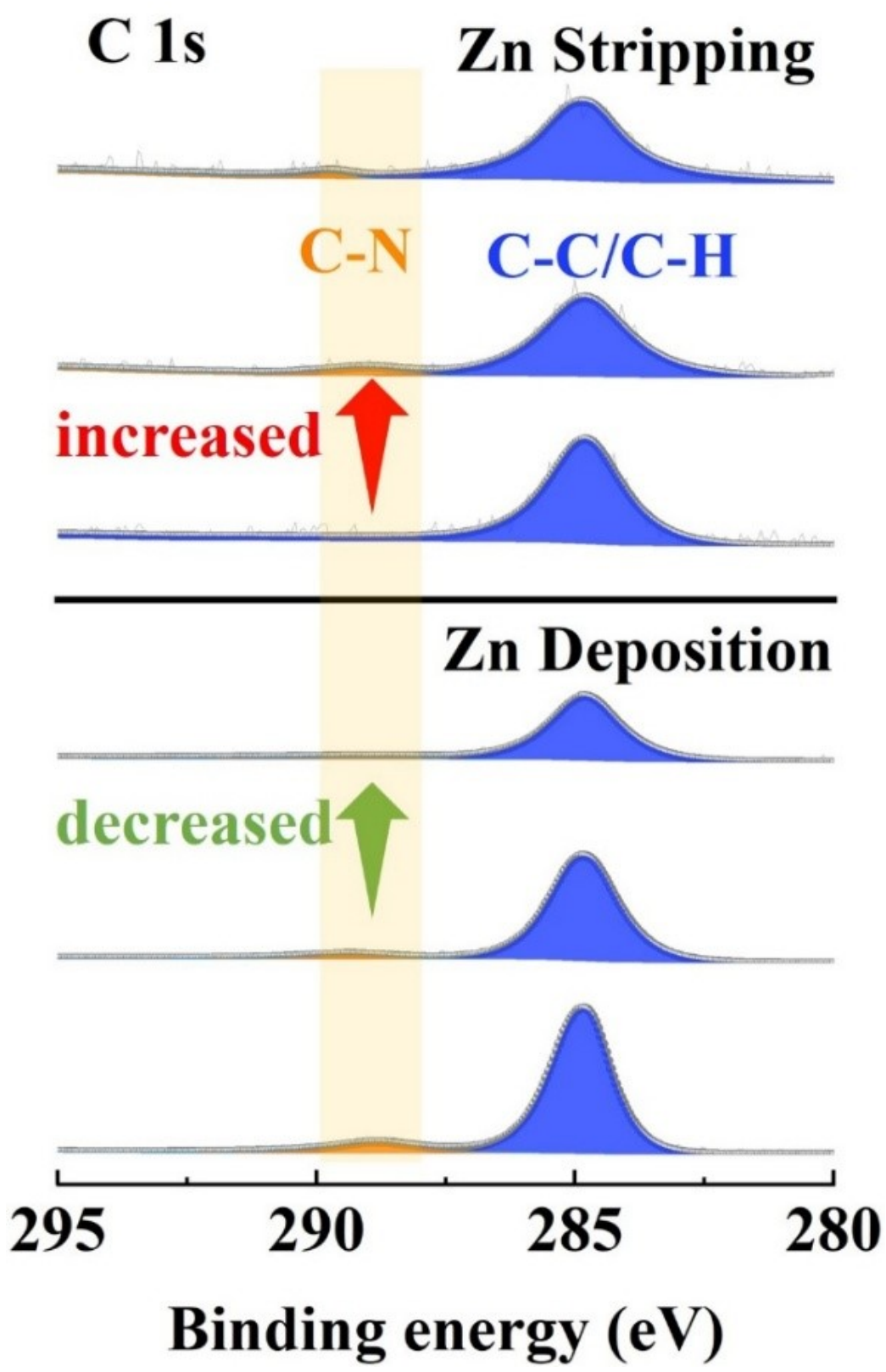

Fig. S7. High-resolution XPS spectra of C 1s during Zn stripping and Zn deposition.

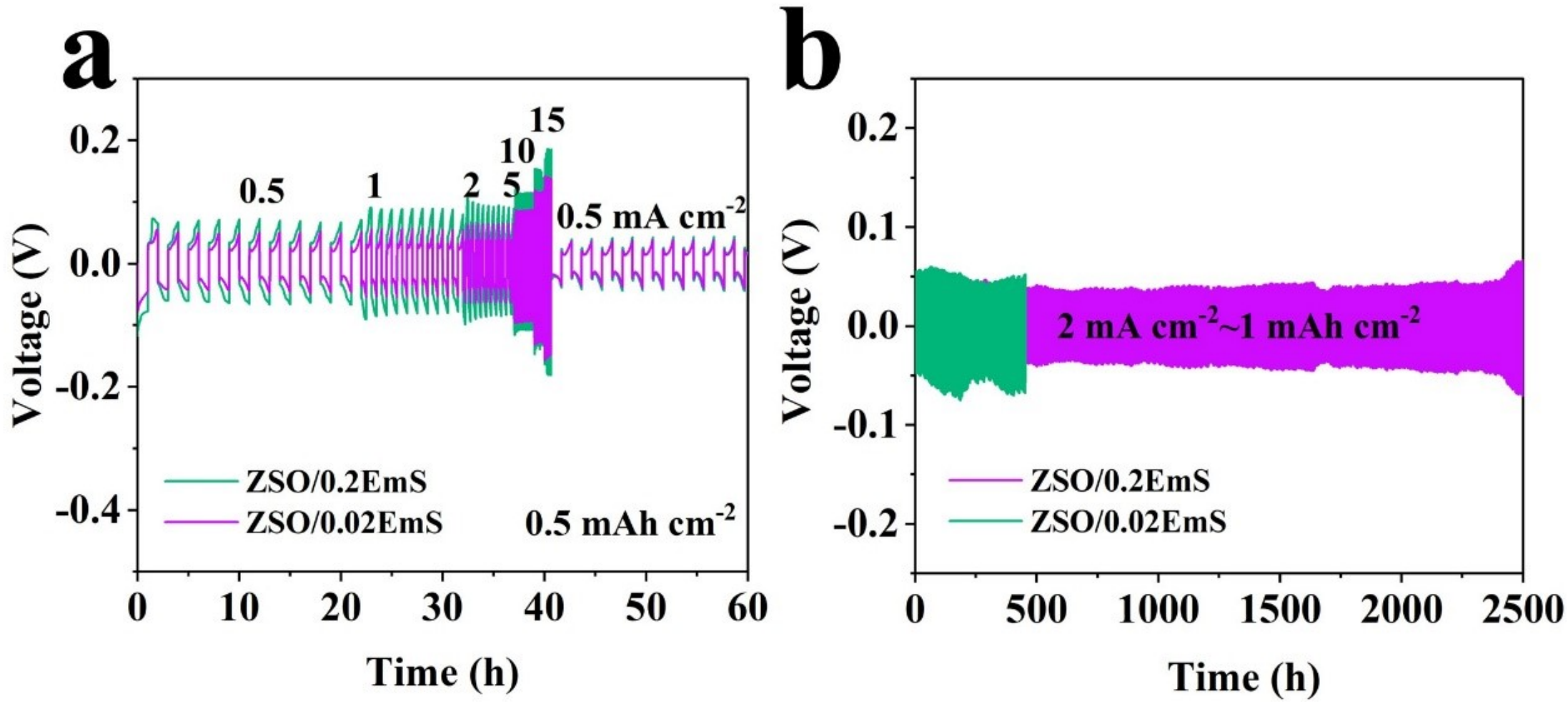

Fig. S8. a) Rate performance and b) cycling performance of Zn//Zn symmetric cells in ZSO/0.02EmS and ZSO/0.2EmS electrolytes.

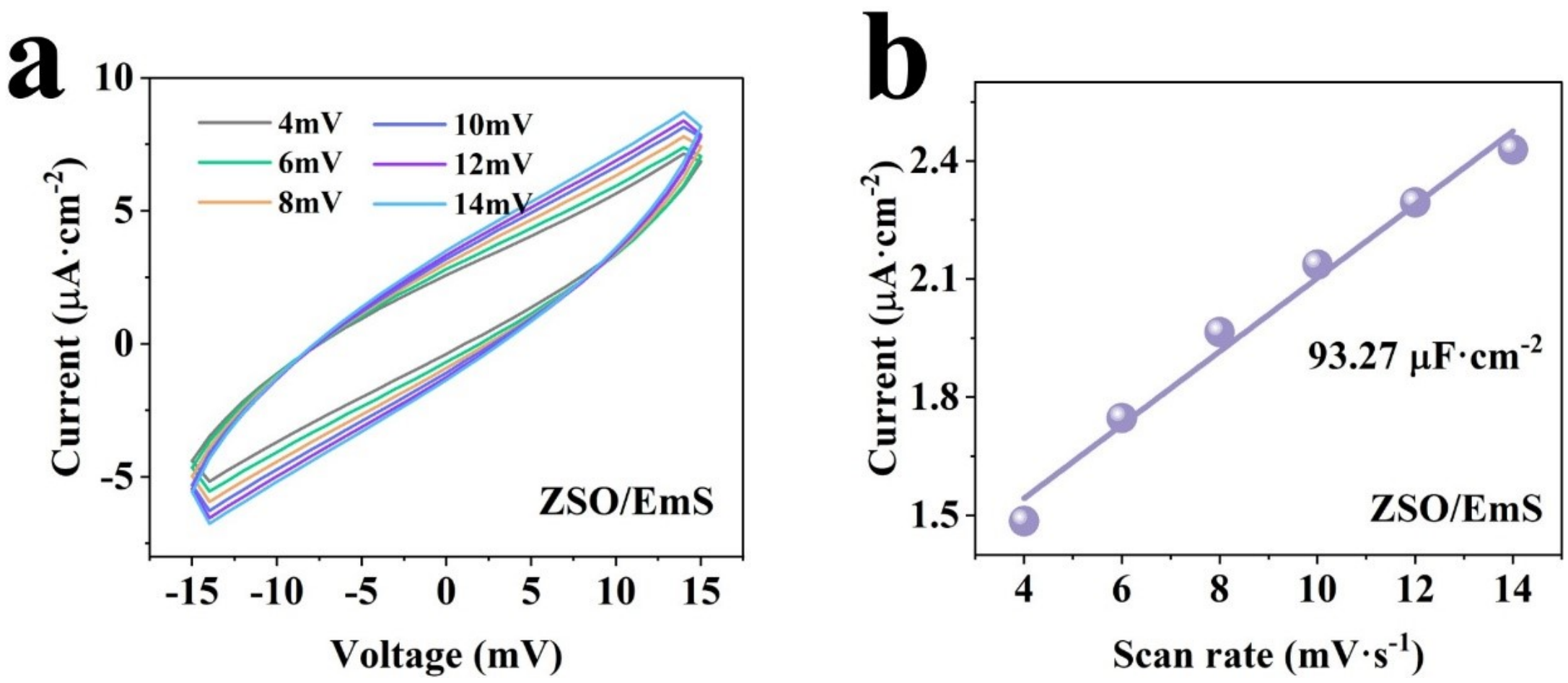

Fig. S9. a) Cyclic voltammograms curves for Zn//Zn cell with ZSO/EmS electrolyte in a voltage range of -15 mV to 15 mV under various scanning rates. b) Plots of capacitive currents versus scan rates.

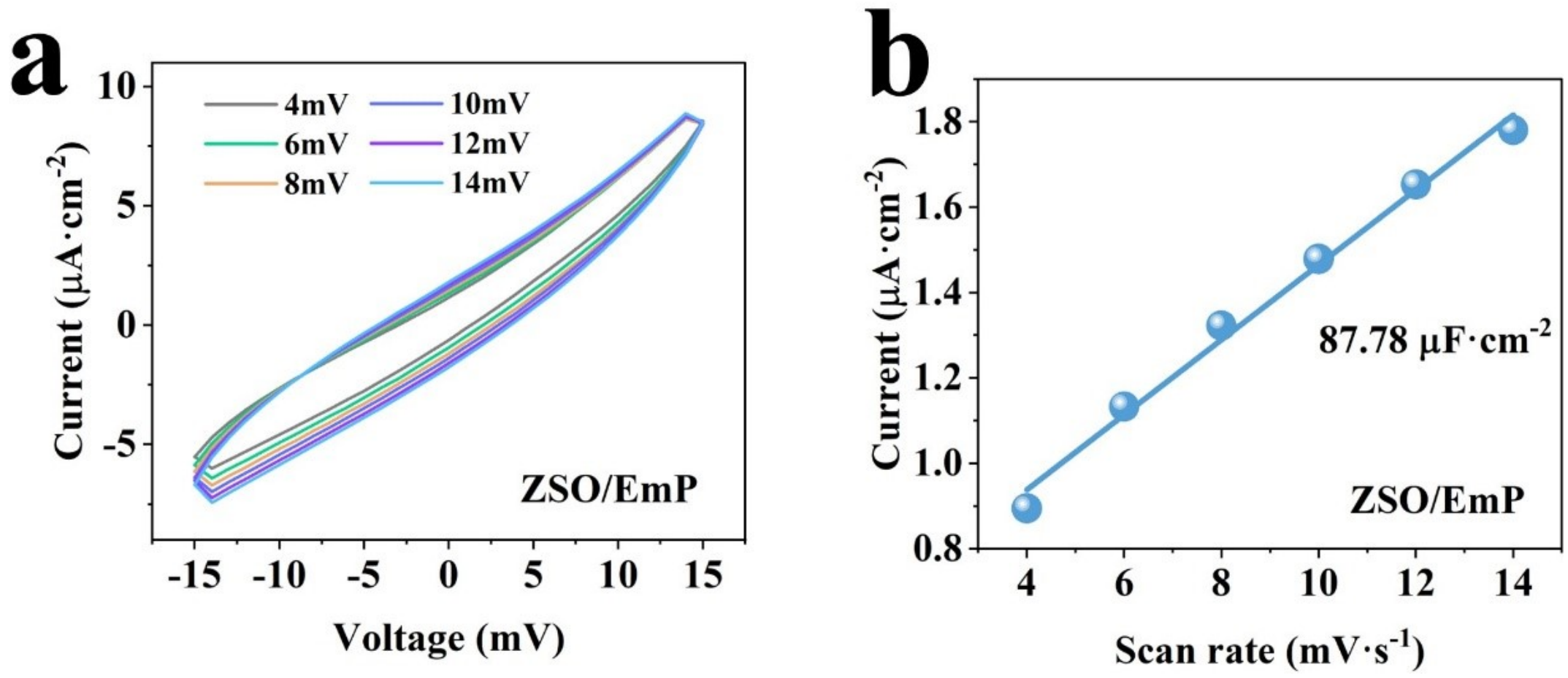

Fig. S10. a) Cyclic voltammograms curves for Zn//Zn cell with ZSO/EmP electrolyte in a voltage range of -15 mV to 15 mV under various scanning rates. b) Plots of capacitive currents versus scan rates.

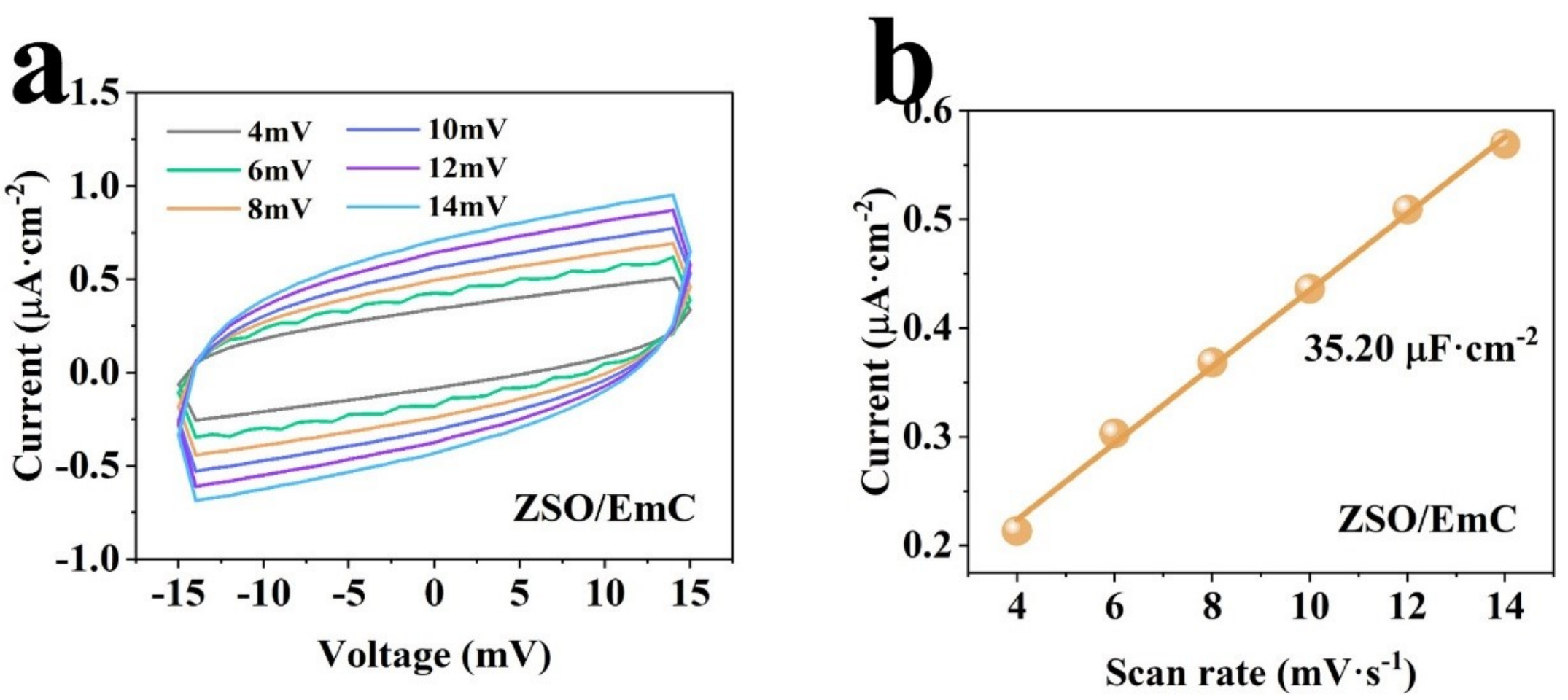

Fig. S11. a) Cyclic voltammograms curves for Zn//Zn cell with ZSO/EmC electrolyte in a voltage range of -15 mV to 15 mV under various scanning rates. b) Plots of capacitive currents versus scan rates.

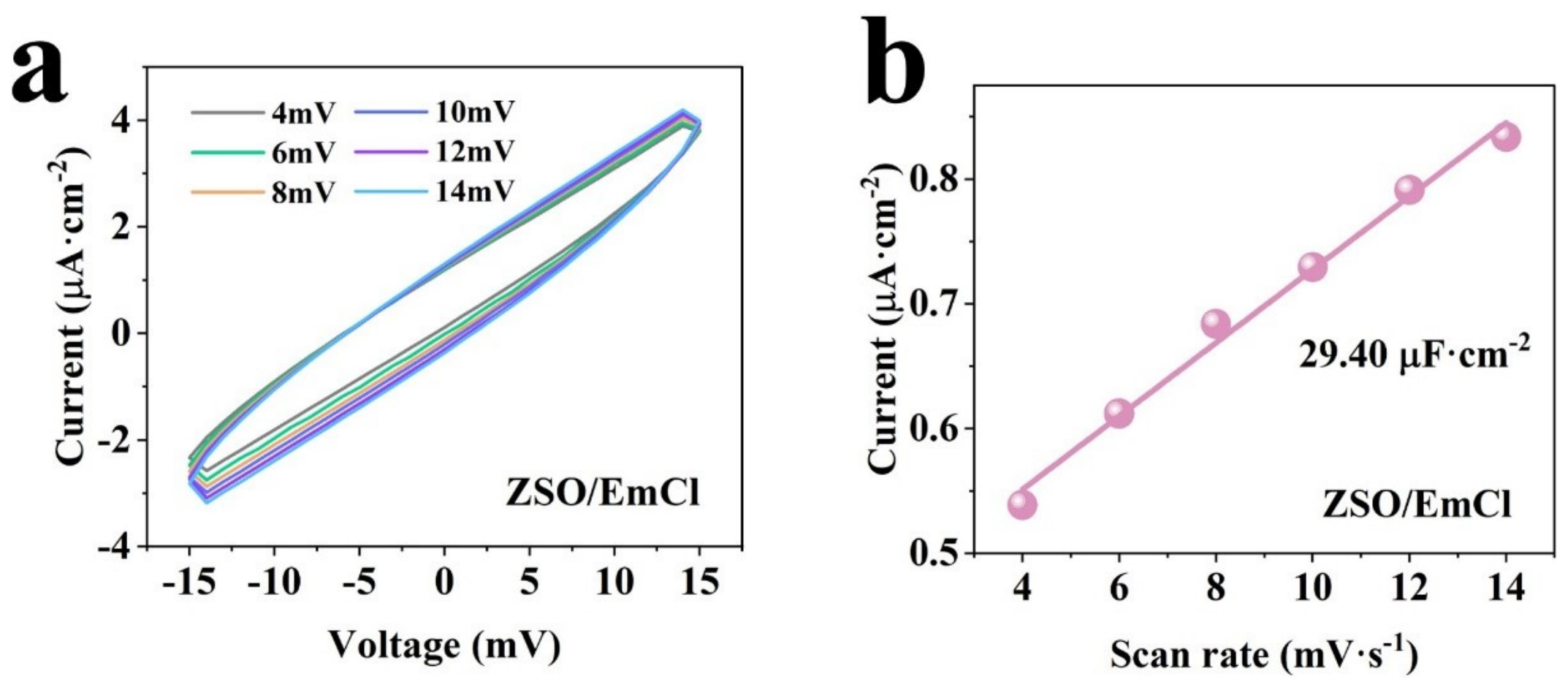

Fig. S12. a) Cyclic voltammograms curves for Zn//Zn cell with ZSO/EmCl electrolyte in a voltage range of -15 mV to 15 mV under various scanning rates. b) Plots of capacitive currents versus scan rates.

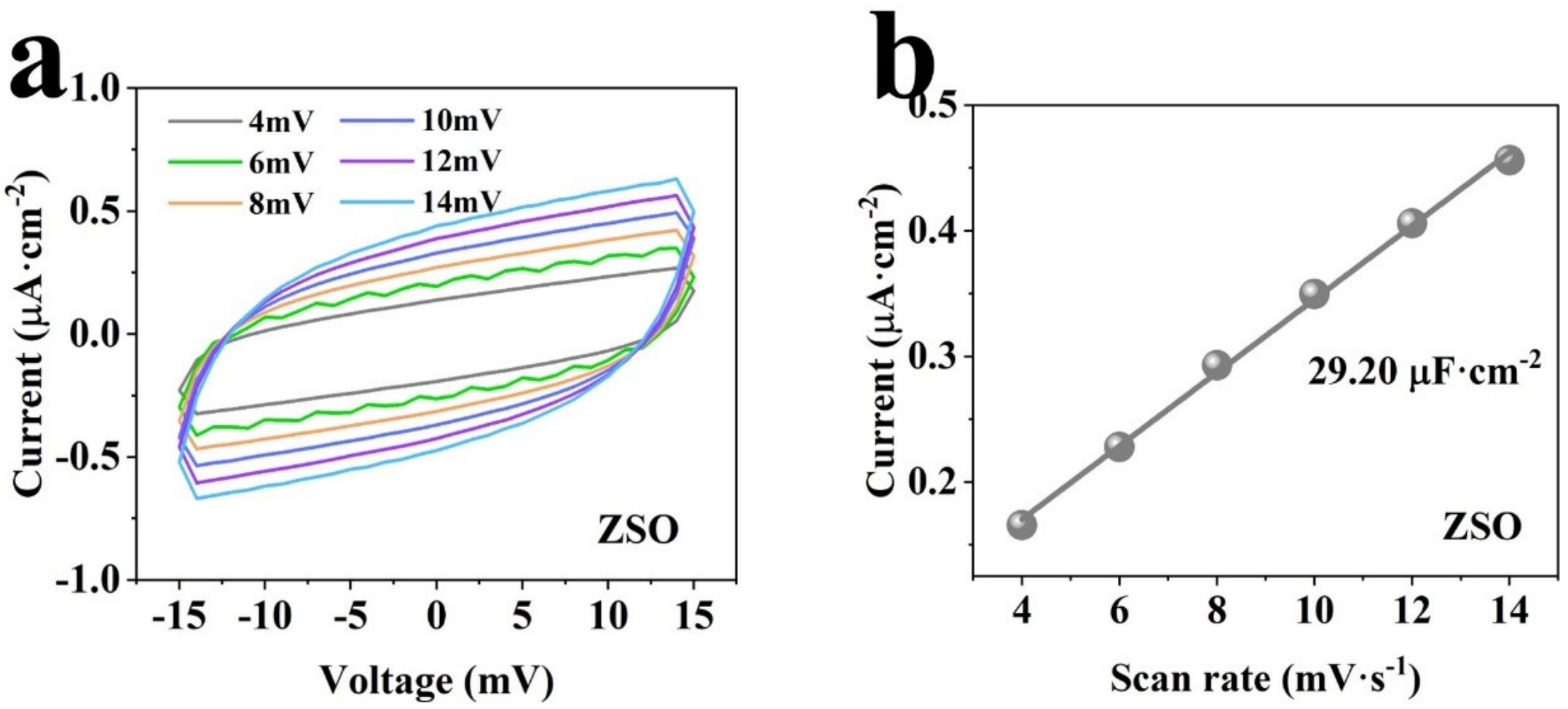

Fig. S13. a) Cyclic voltammogram curves for Zn//Zn cell with ZSO electrolyte in a voltage range of -15 mV to 15 mV under various scanning rates. b) Plots of capacitive currents versus scan rates.

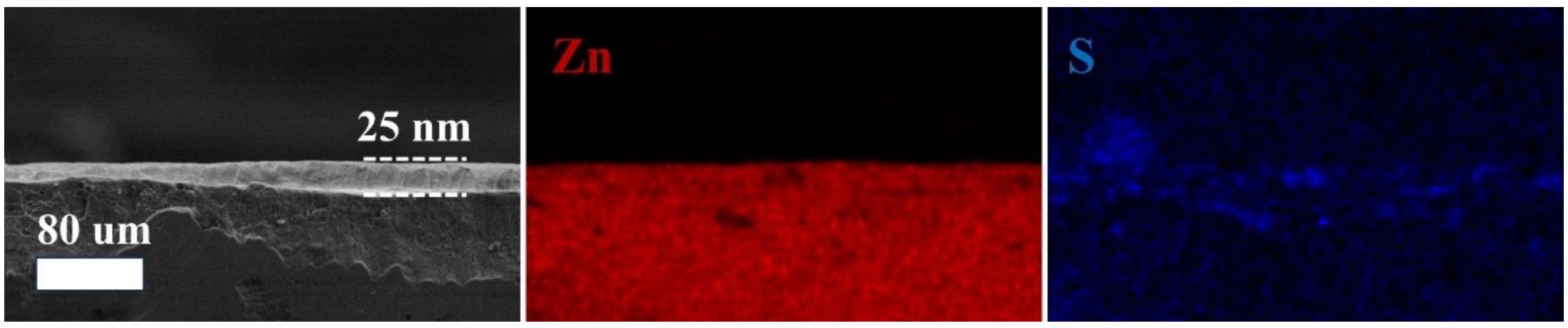

Fig. S14. ZSO/EmS electrolytes at a current density of 1.0 mA cm^{-2} for 60 min.

Table S1. Performance comparison of achieved cycling performance with previous reports in the literatures.

Electrolyte additives	Runtime/Current density ~ Cut-off capacity (mA cm^{-2} ~ mAh cm^{-2})	Ref
1-Ethyl-3-methylimidazolium methanesulfonate	9000h/2.0~1.0	This work
Stevia	8800h/0.5~0.5	Angew. Chem. Int. Ed. 2026, 65, e26100[S1]
N-aminocarbonylmethyl ethanesulfonic acid	760h/50~25	Adv. Energy Mater., 2026, e70829[S2]
I^-/soybean peptide molecules	3200h/2.0~2.0	Adv. Funct. Mater., 2026, e74642[S3]
Disodium naphthalenedisulfonate	7200h/4.0~1.0	Energy Environ. Sci., 2025, 18, 1282-1293[S4]
N-Hydroxyphthalimide	5800h/1.0~1.0	Angew. Chem. Int. Ed. 2025, 64, e202506984[S5]
Pyrrolidone carboxylate sodium	5200h/5.0~5.0	Energy Environ. Sci., 2025, 18, 4416-4430[S6]
Adipic dihydrazide	2400h/1.0~1.0	Chem. Eng. J. 2025, 525, 170207[S7]
Choline phosphate	1100h/1.0~1.0	Energy Storage Mater. 2024, 67, 103299[S8]
Zinc acetylacetonate	1500h/20.0~1.0	Adv. Mater. 2024, 36, 2408706[S9]
ZnSO4:CH_3COONH_4	2400h/2.0~1.0	Adv. Sci. 2022, 9, 2201433[S10]
Cellobiose	4000h/2.0~1.0	Adv. Funct. Mater. 2026, 36,

		e23753[S11]
KH_2PO_4	2400h/2.0~1.0	Adv. Sci., 2026, e22338[S12]

SReferences

S1. J. Wang, H. Zhang, M. Yin, H. Ren, J. Liu, N. Zhang, L. Wang, C. Jiang, T. Chen, B. Cong, D. Wang, C. Wu, H. Liu, S. Dou, D. Chao and B. Wang, Biomimetic anchor-capture effect of multidentate electrolyte additive for ultrastable aqueous zinc ion batteries, *Angew. Chem. Int. Ed.*, 2026, **65**, e26100.

S2. D. Wang, J. Zeng, Y. Tang, K. Dong, X. Wang, Z. Zhang, H. Peng and G. Ma, Engineering a gradient zwitterionic interphase and dehydrated solvation shell for dendrite-free Zn anodes, *Adv. Energy Mater.*, 2026, e70829.

S3. L. Yang, Y. Xu, T. Li, S. Zhang, H. Ma, Y. Shen and T. Lin, Structure-kinetic coordination for reconstructing anode-molecular interfaces toward stable and efficient aqueous zinc-ion batteries, *Adv. Funct. Mater.*, 2026, DOI: 10.1002/adfm.74642, e74642.

S4. H. Lin, L. Zeng, C. Lin, J. Wu, H. He, C. Huang, W. Lai, P. Xiong, F. Xiao, Q. Qian, Q. Chen and J. Lu, Interfacial regulation via configuration screening of a disodium naphthalenedisulfonate additive enabled high-performance wide-pH Zn-based batteries, *Energy Environ. Sci.*, 2025, **18**, 1282-1293.

S5. Y. Sang, J. Wang, M. Xu, B. Zhang, Q. Huang, D. Wang, C. Liu, Y. Zhang, H. Dou and Z. Chen, Engineering robust hydrophilic-hydrophobic interface via π-electron delocalization for ultralong-lived zinc-ion batteries, *Angew. Chem. Int. Ed.*, 2025, **64**, e202506984.

S6. K. Ouyang, S. Chen, L. Yu, H. Qin, A. Liu, Y. Liu, Q. Wu, B. Ran, S. Wei, F. Gao, K. Zhang, J. Hu and Y. Huang, An electrochemically paralleled biomass electrolyte additive facilitates the integrated modification of multi-dimensional Zn metal batteries, *Energy Environ. Sci.*, 2025, **18**, 4416-4430.

S7. X. Zhou, X. Wu and Y. Bando, Highly reversible zinc ion batteries through acylhydrazine modulated electrolyte additives at variable work temperatures,

Chem. Eng. J., 2025, **525**, 170207.

S8. Q. Yan, Z. Hua, Z. Liu, F. Wu, Y. Zhao, R. Chen and L. Li, Synergistic interaction between amphiphilic ion additive groups for stable long-life zinc ion batteries, *Energy Storage Mater.*, 2024, **67**, 103299.

S9. X. Xiao, X. Ye, Z. Wu, X. Wu, J. Yu, L. Gu and S. Liu, Trace small molecular/nano-colloidal multiscale electrolyte additives enable ultra-long lifespan of zinc metal anodes, *Adv. Mater.*, 2024, **36**, 2408706

S10. C. Lin, X. Yang, P. Xiong, H. Lin, L. He, Q. Yao, M. Wei, Q. Qian, Q. Chen and L. Zeng, High-Rate, Large Capacity, and Long Life Dendrite-Free Zn Metal Anode Enabled by Trifunctional Electrolyte Additive with a Wide Temperature Range, *Adv. Sci.*, 2022, **9**, 2201433.

S11. S. Zhang, S. Huang, W. Chen, Z. Hu, D. Min, J. Kim, H. Fu, Y. Yun, W. Cho, B. Kim, B. Li, C. Li, J. Sun and H. Park, Geometrically-Screened, Sterically-Hindered Additive for Wide-Temperature Aqueous Zinc-Ion Batteries, *Adv. Funct. Mater.*, 2026, **36**, e23753.

S12. K. Sun, Y. Geng, S. Gong, F. Li, L. Li, X. Jia, D. Chao and C. Wang, A functionality-graded cathode electrolyte interphase enables ultra-long cycling stability in aqueous Zn-Mn batteries, *Adv. Sci.*, 2026, DOI: 10.1002/advs.202522338, e22338.
